# CROPPER: a metagene creator resource for cross-platform and cross-species compendium studies

**DOI:** 10.1186/1471-2105-7-418

**Published:** 2006-09-22

**Authors:** Jussi Paananen, Markus Storvik, Garry Wong

**Affiliations:** 1 Department of Neurobiology, A. I. Virtanen Institute for Molecular Sciences, P.O. Box 1627, 70211 Kuopio, Finland; 2 Department of Computer Science, University of Kuopio, P.O. Box 1627, 70211 Kuopio, Finland; 3 Department of Biochemistry, University of Kuopio, P.O. Box 1627, 70211 Kuopio, Finland

## Abstract

**Background:**

Current genomic research methods provide researchers with enormous amounts of data. Combining data from different high-throughput research technologies commonly available in biological databases can lead to novel findings and increase research efficiency. However, combining data from different heterogeneous sources is often a very arduous task. These sources can be different microarray technology platforms, genomic databases, or experiments performed on various species. Our aim was to develop a software program that could facilitate the combining of data from heterogeneous sources, and thus allow researchers to perform genomic cross-platform/cross-species studies and to use existing experimental data for compendium studies.

**Results:**

We have developed a web-based software resource, called CROPPER that uses the latest genomic information concerning different data identifiers and orthologous genes from the Ensembl database. CROPPER can be used to combine genomic data from different heterogeneous sources, allowing researchers to perform cross-platform/cross-species compendium studies without the need for complex computational tools or the requirement of setting up one's own in-house database. We also present an example of a simple cross-platform/cross-species compendium study based on publicly available Parkinson's disease data derived from different sources.

**Conclusion:**

CROPPER is a user-friendly and freely available web-based software resource that can be successfully used for cross-species/cross-platform compendium studies.

## Background

Novel genomic research methods have enabled researchers to perform high-throughput experiments that results in massive amounts of experimental data. Combining data from different experiments allows researchers to validate their results and to gain a better understanding of the biological questions being studied. In cross-species studies, data derived from experiments performed on different organisms are combined to find universal themes. In cross-platform studies, common biological questions are studied using different research platforms and technologies. The ability to combine experimental data is particularly useful when extended to combine data available on public data repositories such as sequence, expression, and literature databases.

The desire to perform large-scale studies that combine experimental results from various sources has led to compendium studies that combine cross-species/cross-platform data in order to obtain a larger perspective on biological questions. Unfortunately, for several reasons, the combining of experimental results is anything but a trivial task. These reasons can be divided into biological and technical challenges. The biological challenges include variances between species, different experimental design/conditions, and lack of knowledge of the underlying biological processes. The technical challenges are caused by differences in how experimental data is stored, presented, and managed. Because of the lack of standards, different research equipment, software, and databases identify and structure data in different and unique ways that make it a challenge to combine data obtained from these heterogeneous sources.

To address these technical challenges and to enable researchers to automate the integration of the genomic data derived from heterogeneous sources, without the need for using complex programming and scripting tools, we have developed a user-friendly web-based software resource called CROPPER. CROPPER can be used to combine datasets from different genomic research platforms such as microarrays, biological databases, and experiments performed on different species. When performing the combining process, associated data can be brought along. This facilitates the import of the resulting dataset by the user into the desired statistical/analytical program for further analysis. CROPPER uses the latest genomic information with respect to identifiers and orthologous genes retrieved from the Ensembl [1] database.

## Implementation

CROPPER is developed using Perl version 5.9.1, Bioperl version 1.4 [2] and Ensembl database API written in Perl [3]. CROPPER runs on a web-server which also acts as an application server. Users can use the web-interface to input their datasets and related parameters to the application server. The Application server processes the data, and if required, queries the database server containing installation of the Ensembl-database for information about data identifiers, orthologous genes, and gene annotations. Information about how the identifiers are linked and how the orthologue predictions have been performed can be found from the Ensembl-website.

CROPPER can be used to combine genomic datasets obtained from various heterogeneous sources. As an input, CROPPER takes datasets as delimited text-files. The delimited text-files can have any kind of column structure, but should include a column with an identifier for each data row. All the external database identifiers found from the Ensembl database can be used, including identifiers for major biological databases (e.g. EMBL, GenBank and Uniprot) and technology providers (e.g. Affymetrix and Agilent).

**Figure 1:**
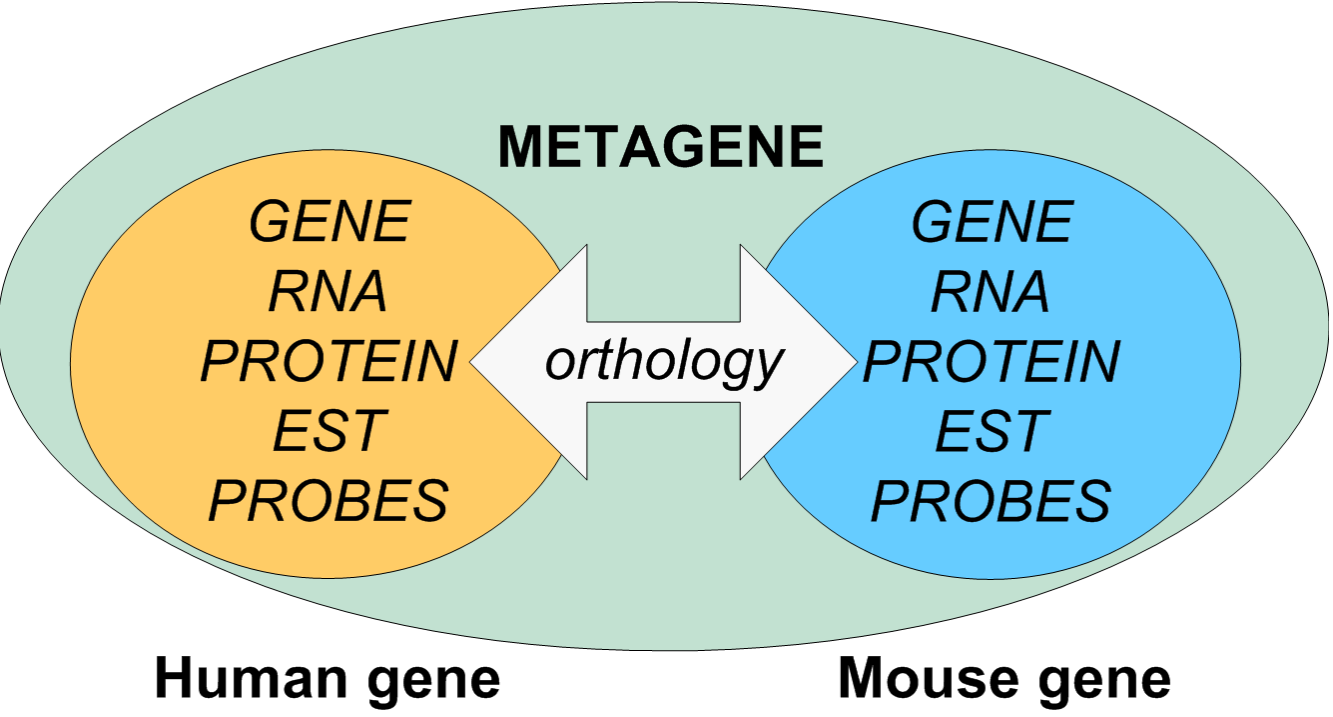
**Concept of metagenes**. A metagene is a common identifier that groups together gene and gene product identifiers originating from a single gene and orthologous genes in other species. Different identifiers can be cross-linked to each other using a common metagene identifier.

The user can select the structure of a result file and choose a metagene identifier to be created for each data row. A metagene identifier is a common identifier automatically created by CROPPER that groups together different identifiers originating from a single gene (these identifiers can be, for example, gene or gene product identifiers, microarray probe identifiers, or identifiers of orthologous genes or products of these genes in other species). For example a gene and a protein coded by an orthologous gene in another species will have a common metagene identifier. The concept of the metagene identifiers is shown in detail in Figure 1.

## Results

CROPPER can be used to combine genomic data obtained from heterogeneous sources. Because CROPPER uses the Ensembl-database for information about data identifiers and orthologous genes, the number of different possible sources of data is enormous. These sources include major technology providers and databases, and therefore CROPPER can be used to perform cross-platform studies using data from these sources. The current Ensembl-build (build 37) contains genomic information from 19 different species (and pre-versions of six additional species) including major model organisms. This allows CROPPER to be used for cross-species studies.

**Figure 2:**
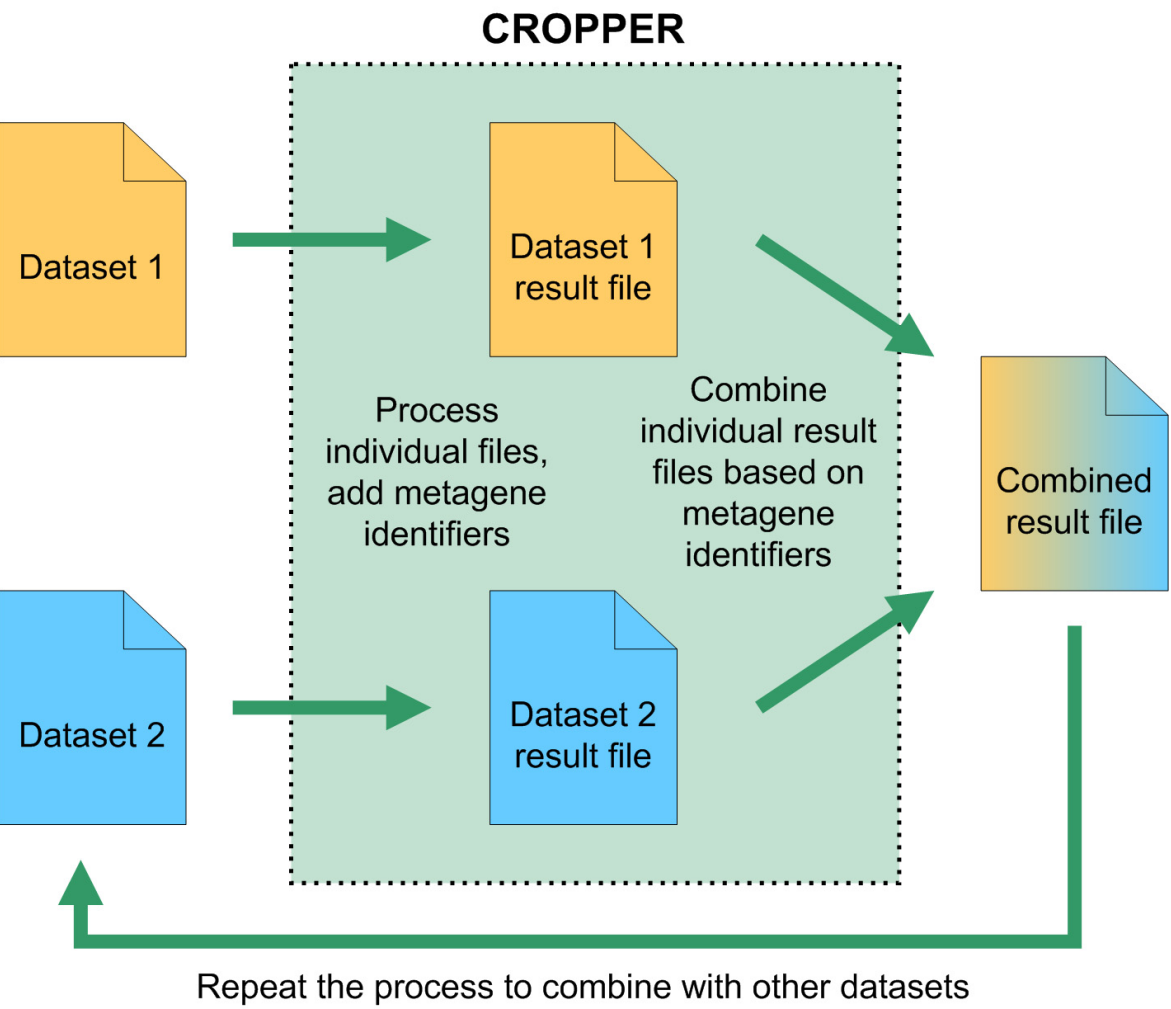
**Process flow of using CROPPER**. Datasets are first processed individually. This processing adds a metagene identifier to each data row. After the processing of both datasets, the datasets can be combined using the metagene identifiers. Result file containing the combined data rows is produced. Additional datasets can be combined to the result file by repeating the process and including the combined result file as the second dataset.

Using CROPPER is a straightforward process which is divided into two parts; processing of individual dataset files and combining the processed files. When processing individual files, users can choose to annotate and re-structure their dataset, and additionally add a metagene identifier for each data row. After processing the individual files and adding metagene identifiers, the processed datasets can then be directly used for analysis in suitable 3^rd ^party analysis software, or alternatively combined with CROPPER using the common metagene identifiers. The combining process produces a result dataset that the users can then import to the analysis software of their choice. The flow of processing and combining datasets using CROPPER is presented in Figure 2.

### Performing an example compendium study

To demonstrate how CROPPER can be used in cross-species/cross-platform compendium studies, we performed a small-scale compendium study. The datasets used were; Mouse Full Powerblot Western Array dataset used for proteomic analysis after Rasagiline treatment (downloaded from GEO, GSE1857), Affymetrix GeneChip Human Genome Focus Array dataset used for gene expression profiling of parkinsonian substantia nigra pars compacta [4], Affymetrix GeneChip *C. elegans *Genome Array dataset used for identification of gene expression changes in transgenic *C. elegans *overexpressing human mutant A53T α-synuclein [5] and Affymetrix GeneChip Human Genome 133A set used for gene expression profiling of MPTP-lesioned macaque model of Parkinson's disease [6]. The reasons for selecting these datasets were the common focus of the studies (neurodegenerative Parkinson's disease), the wide variety of covered species (*Homo sapiens*, *Caenorhabditis elegans*, *Mus musculus *and *Macaca fascicularis*) and differences in used platforms (protein and gene expression arrays). The question we wanted to study was: are there common themes between the human disease state and animal disease models? Moreover, what are the themes that can be found from genes with altered expression in animal models, but not in the humans?

CROPPER was successfully used to assign metagene identifiers to the datasets and then to combine the datasets in to a single result dataset, which was used in further analyses (see Table 1 for example of the combined result dataset). Z-transformation [7] was used to normalise the data by calculating z-ratios for the difference between control and treatment data in each of the original study cases (see Additional file 1 for the complete combined result dataset with calculated z-ratios).

**Table 1:** Example of combined result dataset

	*Homo sapiens*		*Mus musculus*		*Caenorhabditis elegans*		*Macaca fascicularis*	
	
**Metagene ID**	**Affymetrix probe ID**	**Gene description**	**Uniprot ID**	**Gene description**	**Wormbase Gene ID**	**Gene description**	**Affymetrix probe ID**	**Gene description**
MGD59E612	216248_s_at	Orphan nuclear receptor NR4A2 (Orphan nuclear receptor NURR1) (Immediate-early response protein NOT) (Transcriptionally-inducible nuclear receptor).	Q06219	nuclear receptor subfamily 4, group A, member 2	C48D5.1	Nuclear hormone receptor family member nhr-6 (Cnr8).	216248_s_at	Orphan nuclear receptor NR4A2 (Orphan nuclear receptor NURR1) (Immediate-early response protein NOT) (Transcriptionally-inducible nuclear receptor).
MGDH20656	200746_s_at		P04901		F13D12.7	Guanine nucleotide-binding protein beta subunit 1.	200746_s_at	Guanine nucleotide-binding protein G(I)/G(S)/G(T) beta subunit 1 (Transducin beta chain 1).
MGDH22681	201533_at	Beta-catenin.	Q02248	catenin (cadherin associated protein), beta 1	K05C4.6	HuMPback (dorsal lumps) family member (hmp-2)	201533_at	Beta-catenin.
MGDH22861	203333_at	Kinesin-associated protein 3 (Smg GDS-associated protein).	P70188	kinesin-associated protein 3	F56C9.1	Putative serine/threonine protein phosphatase F56C9.1 in chromosome III (EC 3.1.3.16).	203333_at	Kinesin-associated protein 3 (Smg GDS-associated protein).
MGDH22922	200075_s_at	Guanylate kinase (EC 2.7.4.8) (GMP kinase).	Q64520	guanylate kinase 1	T03F1.8		200075_s_at	Guanylate kinase (EC 2.7.4.8) (GMP kinase).
MGDH23987	217746_s_at	Programmed cell death 6-interacting protein (PDCD6-interacting protein) (ALG-2-interacting protein 1) (Hp95).	O88695		R10E12.1	Apoptosis-linked gene 2 interacting protein X 1 (Protein pqn-58) (Protein YNK1).	217746_s_at	Programmed cell death 6-interacting protein (PDCD6-interacting protein) (ALG-2-interacting protein 1) (Hp95).
MGDH2564	203087_s_at	Kinesin-like protein KIF2 (Kinesin-2) (HK2).	P28740	kinesin family member 2A	K11D9.1	Kinesin-Like Protein family member (klp-7)	213598_at	Kinesin-like protein KIF2 (Kinesin-2) (HK2).
MGDH2591	209503_s_at	26S protease regulatory subunit 8 (Proteasome subunit p45) (p45/SUG) (Proteasome 26S subunit ATPase 5) (Thyroid hormone receptor-interacting protein 1) (TRIP1).	P47210		Y49E10.1	proteasome Regulatory Particle, ATPase-like family member (rpt-6)	209503_s_at	26S protease regulatory subunit 8 (Proteasome subunit p45) (p45/SUG) (Proteasome 26S subunit ATPase 5) (Thyroid hormone receptor-interacting protein 1) (TRIP1).
MGDH26335	201390_s_at	Casein kinase II subunit beta (CK II beta) (Phosvitin) (G5a).	P13862		T01G9.6	Casein kinase II beta subunit (CK II beta).	201390_s_at	Casein kinase II subunit beta (CK II beta) (Phosvitin) (G5a).
MGDH2988	207614_s_at	Cullin-1 (CUL-1).	Q9WTX6	cullin 1	D2045.6	Cullin-1 (Abnormal cell lineage 19 protein).	207614_s_at	Cullin-1 (CUL-1).
MGDH6882	200864_s_at		P24410		F53G12.1	RAB family member (rab-11.1)	200864_s_at	Ras-related protein Rab-11A (Rab-11) (YL8).
MGDH8694	201220_x_at		P56546	C-terminal binding protein 2	F49E10.5		210835_s_at	C-terminal-binding protein 2 (CtBP2).

CROPPER was used to combine datasets from four distinct experiments. Metagene identifiers were assigned to each data row. Inspection of the gene descriptions reveals that it is likely that combining has been successfully and metagene identifiers have been assigned to a common group of genes and gene products across the different species and technology platforms.

**Figure 3:**
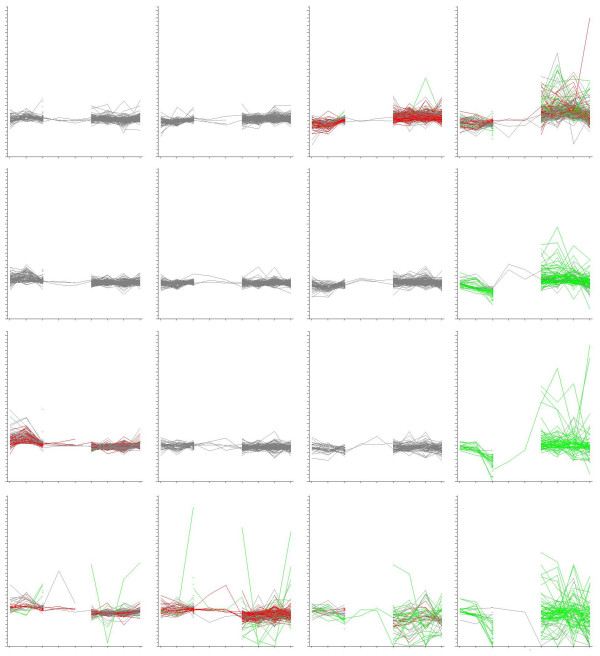
**SOM Clustering of data combined using CROPPER**. Four different Parkinson's disease datasets were combined by aligning the metagenes with CROPPER. The data consisted of a total of 9 conditions originating from the datasets. The conditions are shown in the ordinate axis and their z-transformed values are shown in the y-axis. For normalization, the differences in the data value distributions were z-transformed. This was followed by calculation of the z-ratio, in which the differences of the z-values of the treated samples were subtracted from z-values of the controls (for method details, see Cheadle et al. 2002 [7]). The limit for significant alteration was z-ratio ± 1 defined as more than one standard deviation in the z-values of control and treatment data points. The gene expression data from human represent 4055 metagenes. These were clustered into 16 clusters using a self-organizing map (SOM). The expression profiles of the metagenes with altered expression in both human and animal data sets were coloured in green. These 247 "green" genes were considered to be candidates for human neurodegenerative diseases. The genes with altered expression only in the animal experiment datasets, but not in the human datasets were coloured in red. The 225 "red" genes may suggest mechanisms in animal neurodegeneration models, but not in human Parkinson's disease. The separation of red and green genes to peripheral clusters indicates good clustering resolution. The lists of "red" and "green" metagenes were further analyzed for the enriched human KEGG and GO terms based on the human gene identifiers corresponding to the assigned metagenes.

The genes that were present in the human dataset (4055 genes) were clustered into 16 clusters using a self-organizing map (SOM) with GeneSpring 7.2 (Agilent Technologies, USA) as presented in Figure 3. A new gene list was created from the clusters in which the expression levels greatly varied between the conditions (1262 genes). From this list, those also regulated in the human disease state (246 genes exceeding Z-ratio of ± 1, defined as the difference between the z-values of the control and treated samples divided by the standard deviation of all differences) were considered to be the most likely candidates in the disease models. The profiles of these 246 genes are marked in green in the figure 3. In addition, the genes which were regulated in any of the animal data sets by a Z-ratio of ± 1, but not in the human Parkinson's disease sample (Z-ratio of >-0.2 and < 0.2) (225 genes total) are marked in red in figure 3. These two lists of genes were inspected for the enriched KEGG-pathways by using DAVID [8] with the whole human genome as a background list and for the enriched GO-terms by using GENERATOR [9] with the present human genes from the combined dataset as a background list. The results from different phases of the analysis are presented in the Additional file 2.

The biological themes discovered from the lists of regulated genes suggest that biological hypotheses with explanatory power can be generated using the metagene approach. The results also suggest that combining datasets from different studies provides a valuable tool for validating results, as the human dataset was used to filter out genes not detected in the human disease state. This makes it possible to detect genes from the animal experiments that are most likely to be involved in the actual human disease, supporting a selection process of candidate genes based not only on statistical power, but also on the biological differences between species. In the analysed data, the clustering of genes based on the GO-terms revealed that the transport proteins, molecular biosynthesis mechanism, and the neurofilaments are good candidates for studies in most of the animal models for neurodegeneration. Calmodulin and calcium related modulatory mechanisms are also detected in the animal models. Downstream data extraction can be performed by export of the combined data to view enriched terms from the KEGG pathway (Additional file 2), that then can be used to create new hypotheses for the further studies.

## Discussion and conclusion

Researchers performing experiments using novel genomic research methods often face the challenge of combining their experimental results with results derived from different heterogeneous sources, such as experiments conducted using different technologies, different model organisms or results retrieved from public databases. We have developed a web-based software program called CROPPER that automates this task.

Several compendium studies that combine data from different sources have been published, but it is common that the actual combination and integration of the data has been done using custom-made software programs and scripts that are useful only for the data used for the specific study [10-12]. This has resulted in the need of complete end-user programs that biologists can use to combine their datasets. Different resources have been developed to address this challenge [13-18], but are hindered by several limitations. These limitations include focusing on a single (or very limited amount) of species/technology, requiring a strict pre-defined format on datasets, not allowing customisation of the result file or including actual experimental data in the dataset. CROPPER differs from these resources by giving users a good flexibility on how to import and export data and broad coverage of all the major databases, technologies and species, making CROPPER useful for a very wide variety of users.

One of the main strengths of CROPPER is that it uses the Ensembl-database, therefore ensuring that all the major data sources and species are covered, and that the data is always up-to-date. What distinguishes CROPPER from the data mining tools provided by Ensembl [19,20], is that CROPPER is specially designed for automated data integration, implementing the original metagene approach, therefore allowing users to combine numerous distinct datasets, bring the experimental data along, and not requiring other software or programming tools to facilitate the combining. This is in addition to its ease of use to help biologists combine their datasets and to gain increased power for their research, which could not be obtained by direct usage of Ensembl data mining tools. Moreover, users do not need to be familiar with Ensembl or their data mining tools to use CROPPER.

When performing compendium studies, the researcher should pay attention to the steps taken in data combining. For example it should be clear that when cross-linking gene datasets to protein datasets, accuracy is lost. It is also lost when combining datasets derived from different technologies. For example, when combining data from cDNA and oligonucleotide microarrays, the actual experimental measurements are very rarely directly comparable. It should be noted that CROPPER only combines the related data rows, but does not alter the experimental data in any way. Therefore it is likely that depending on the type of the study, different methods and tools are required to make the data comparable. In many cases, the Z-ratio method presented here will work, but other methods can also be used. Many statistical and computational methods and software are publicly or commercially available towards this end. When performing compendium studies, the combining of data elements is usually the first and most difficult task and using CROPPER helps researchers to overcome this major bottle-neck in the integration of genomic data.

## Availability and requirements

**Project name**: CROPPER

**Project homepage**: 

**Operating system(s)**: Platform independent

**Programming language**: Perl

**Other requirements**:

**License**: Free for academic use

**Any restrictions to use by non-academics**: License needed

## Authors' contributions

JP designed and developed the methodology and software and drafted the manuscript. MS performed the compendium study and helped to draft the manuscript. GW conceived the study, participated in its design and coordination and helped to draft the manuscript. All authors read and approved the final manuscript.

## Supplementary Material

Additional File 1 **Combined data used for the example analysis**. Combined data from the four different neurodegenerative studies, used for the example compendium study. Data presented with z-ratios (treatment vs. the absolute control) and the genes regulated in human and/or in other organisms are presented. Click here for file

Additional File 2 **The biological themes and patterns detected in the combined data**. First worksheet contains the enriched KEGG pathways, number of genes with association to a pathway, and the p-value for the statistical significance. Second and third worksheet contain the enriched GO terms detected in the combined data, clustered into 1, 2 and 3 clusters based on the associated GO terms. Click here for file
